# Capecitabine and stereotactic radiation in the management of breast cancer brain metastases

**DOI:** 10.1186/s12885-021-08302-9

**Published:** 2021-05-15

**Authors:** Matthew N. Mills, Afrin Naz, Chetna Thawani, Chelsea Walker, Nicholas B. Figura, Sergiy Kushchayev, Daniel E. Oliver, Arnold B. Etame, Hsiang-Hsuan Michael Yu, Timothy J. Robinson, James K. C. Liu, Michael A. Vogelbaum, Peter A. Forsyth, Brian J. Czerniecki, Hatem H. Soliman, Hyo S. Han, Kamran A. Ahmed

**Affiliations:** 1 Department of Radiation Oncology, H. Lee Moffitt Cancer Center and Research Institute, 12902 Magnolia Dr, Tampa, FL 33612 USA; 2 Morsani College of Medicine, University of South Florida, Tampa, FL 33612 USA; 3 Department of Radiology, H. Lee Moffitt Cancer Center and Research Institute, Tampa, FL 33612 USA; 4 Department of Neuro Oncology, H. Lee Moffitt Cancer Center and Research Institute, Tampa, FL 33612 USA; 5 Department of Breast Oncology, H. Lee Moffitt Cancer Center and Research Institute, Tampa, FL 33612 USA

**Keywords:** Capecitabine, Xeloda, Breast cancer, Brain metastases, Stereotactic radiotherapy

## Abstract

**Background:**

Little is known about the safety and efficacy of concurrent capecitabine and stereotactic radiotherapy in the setting of breast cancer brain metastases (BCBM).

**Methods:**

Twenty-three patients with BCBM underwent 31 stereotactic sessions to 90 lesions from 2005 to 2019 with receipt of capecitabine. The Kaplan-Meier method was used to calculate overall survival (OS), local control (LC), and distant intracranial control (DIC) from the date of stereotactic radiation. Imaging was independently reviewed by a neuro-radiologist.

**Results:**

Median follow-up from stereotactic radiation was 9.2 months. Receptor types of patients treated included triple negative (*n* = 7), hormone receptor (HR)+/HER2- (*n* = 7), HR+/HER2+ (*n* = 6), and HR−/HER2+ (*n* = 3). Fourteen patients had stage IV disease prior to BCBM diagnosis. The median number of brain metastases treated per patient was 3 (1 to 12). The median dose of stereotactic radiosurgery (SRS) was 21 Gy (range: 15–24 Gy) treated in a single fraction and for lesions treated with fractionated stereotactic radiation therapy (FSRT) 25 Gy (24–30 Gy) in a median of 5 fractions (range: 3–5). Of the 31 stereotactic sessions, 71% occurred within 1 month of capecitabine. No increased toxicity was noted in our series with no cases of radionecrosis. The 1-year OS, LC, and DIC were 46, 88, and 30%, respectively.

**Conclusions:**

In our single institution experience, we demonstrate stereotactic radiation and capecitabine to be a safe treatment for patients with BCBM with adequate LC. Further study is needed to determine the potential synergy between stereotactic radiation and capecitabine in the management of BCBM.

## Background

Breast cancer brain metastases (BCBM) have become an increasingly common diagnosis in advanced breast cancer [1]. Triple negative and HER2+ breast cancers have been identified as risk factors for the development of brain metastases [2–4]. Local therapies including surgical resection and radiation therapy continue to be mainstays in the management of brain metastases.

Given the concurrent extracranial disease burden in brain metastatic patients, systemic therapies are often advised which may also have an impact on intracranial brain control [1]. Capecitabine is an oral prodrug of 5-flourouracil (5-FU), an antimetabolite, and undergoes 3-step enzymatic conversion to 5-FU. While capecitabine does not cross the intact blood-brain-barrier (BBB), it has demonstrated intracranial penetration in surgically resected BCBM samples, presumably due to the defective BBB that is associated with contrast enhancement [5]. In the management of HER2+ BCBM, capecitabine has been combined with the HER tyrosine kinase inhibitor (TKI) lapatinib demonstrating a central nervous system (CNS) response rate of 66% [6]. The HER TKI neratinib demonstrated activity against HER2-positive BCBM with an objective response rate of 49% in lapatinib naive patients [7]. Capecitabine alone is commonly prescribed in the adjuvant setting in triple negative tumors following results of the CREATE-X trial which demonstrated the rate of disease-free survival as 69.8% in the capecitabine group versus 56.1% in the control group and improved overall survival (OS) [8].

Capecitabine and 5-FU are known radiosensitizers and are commonly used alongside radiation therapy to enhance the effect of therapy. This is commonly the case in gastrointestinal malignancies such as rectal, pancreatic, and anal cancers [9–11]. Care must be taken to avoid the late effects of radiation therapy in the management of brain metastases with the most worrisome side effect being radionecrosis [12]. Given the known effects of capecitabine as a radiosensitizer, its known role in the management of BCBM, and its increasingly common use in the systemic management of triple negative breast cancer, we conducted a retrospective analysis to assess adverse events of combined therapy as well as potential for a synergistic effect with concurrent treatment.

## Methods

BCBM patients were identified from our prospectively maintained database of patients receiving radiation therapy. Patients were included if they were diagnosed with brain metastases that were treated with stereotactic radiation within 6 months of receiving capecitabine (either before, during or after administration). Patients underwent stereotactic radiosurgery (SRS) or fractionated stereotactic radiotherapy (FSRT) between January 2005 and November 2019 and were followed until February 2020. The study was approved by the University of South Florida Institutional Review Board. All methods were carried out in accordance with relevant guidelines and regulations.

### Stereotactic radiation technique

Stereotactic radiation technique was conducted as previously described [13, 14]. Brain metastases were assessed using magnetic resonance imaging (MRI) (Siemens Sonata, Siemens Medical Systems, Erlangen, Germany) with 1 mm slices for treatment planning purposes prior to the delivery of radiation. The MRI image was co-registered and fused with computed tomography simulation (CT) imaging (General Electric Medical System, Milwaukee, WI). Patient immobilization was achieved by using a commercially available head mask fixation system (BrainlabAG, Feldkirchen, Germany). A uniform 1–2 mm expansion of the gross tumor volume (GTV) was used to create the planning target volume (PTV). All BCBMs were treated with SRS in a single session except 10 metastases treated with FSRT. Seven lesions (8%) underwent prior surgery. Doses were prescribed to ensure coverage of at least 95% of the PTV with the prescription dose. Treatments were delivered using multiple dynamic conformal arcs or intensity modulated radiotherapy (IMRT). Image guidance was provided with the BrainLab ExacTrac positioning system.

### Follow-up

Patients in this study were followed with examinations by the treating radiation oncologist, neurosurgeon, and/or medical oncologist and with MRI imaging at 2–3-month intervals [13]. At each visit neurologic status was assessed. Local brain metastasis failure was defined by RANO-BM criteria [15] that remained consistent or demonstrated continued progression on subsequent imaging whereas local brain metastases control (LC) included all treated lesions not meeting this definition. Distant brain metastases failure was defined as new brain metastases or leptomeningeal enhancement outside the previously irradiated field. Distant intracranial control (DIC) was defined as freedom from development of brain metastases or leptomeningeal disease outside of the irradiated field. Imaging was independently reviewed by a neuro-radiologist (SK). OS was calculated from the date of stereotactic radiation and the date of BCBM diagnosis to the date of death.

### Statistical analysis

Statistical analyses were performed using JMP 13 (SAS Institute Inc., Cary, NC, USA). Descriptive statistics were used to summarize the cohort including median and range for continuous variables or counts and percentages for categorical variables. The local and DIC rates, as well as OS were calculated from the date of BCBM diagnosis or radiation treatment to the date of progression or death using the Kaplan–Meier (KM) method, with the log-rank test used to test differences between groups.

## Results

### Patient and treatment characteristics

Patient and treatment characteristics are described in Table 1. A total of 23 patients treated over 31 treatments sessions to 90 BCBM lesions were identified. The majority of patients had invasive ductal carcinoma (*n* = 21; 91%) with 1 patient with invasive lobular carcinoma, and 1 patient with metaplastic carcinoma (each 4%). Fourteen patients (61%) had stage IV disease prior to BCBM diagnosis. Median follow-up from the date of brain metastases diagnosis was 20.2 months (range: 5.5–96.4 months). Twenty-two stereotactic sessions (71%) occurred within 1 month of the receipt of capecitabine. Stereotactic radiation was delivered concurrently with capecitabine in many of the treatment sessions (*n* = 15; 48%). Stereotactic radiation was delivered before or after capecitabine in 39% (*n* = 12) and 13% (*n* = 4) treatment sessions, respectively. In patients not treated concurrently, the median time between receipt of capecitabine and stereotactic radiation was 1.5 months (range: 0.26–5.5 months). Receptor types of patients treated were 30% (*n* = 7) triple negative, 30% (*n* = 7) HR+/HER2-, 26% (*n* = 6) HR+/HER2+, and 3 (*n* = 13%) HER2 + .

**Table 1:** Patient and Treatment Characteristics

Variable	n	%
**No. of Patients**	23	
**Treatment Sessions**	31	
**No. of Lesions**	90	
**F/U from RT (months)**		
Median (range)	9.2 (1.9–94.1)	
**F/U from Brain Metastases Diagnosis (months)**		
Median (range)	20.2 (5.5–96.4)	
**Age at time of RT**		
Median (range)	56 (40–75)	
**KPS**		
100	7	30%
90	8	35%
80	7	30%
60	1	4%
**Lesions Treated Per Patient**		
Median (range)	3 (1–12)	
**Receptor Status**		
HR+/HER2-	7	30%
HR- /HER2+	3	13%
HR+/HER2+	6	26%
HR−/HER2-	7	30%
**RT in Relation to Capecitabine**		
Before	12	39%
After	4	13%
Concurrent	15	48%
**Interval Between Capecitabine and RT (months)**		
Median (range)	1.5 (0.26–5.5)	
**Concurrent Therapy with Capecitabine**		
None	7	30%
Additional Chemotherapy	8	35%
Trastuzumab	2	9%
Trastuzumab + Chemotherapy	2	9%
Trastuzumab + TKI	2	9%
Pembrolizumab	1	4%
TKI	1	4%

Abbreviations: *F/U* follow-up, *RT* radiation therapy, *KPS* Karnofsky performance status, *TKI* tyrosine kinase inhibitor

Radiation details are described in Table 2. The median PTV of lesions was 0.71 cm^3^ (range: 0.01–39.1 cm^3^). The median dose of SRS was 21 Gy (range: 15–24 Gy) treated in a single fraction and for lesions treated with FSRT 25 (24–30 Gy) in a median of 5 fractions (range: 3–5). Seven lesions (8%) were treated post-operatively.

**Table 2:** Radiation Treatment Details

Variable	n	%
**Technique**		
SRS	80	89%
FSRT	10	11%
**SRS Dose (Gy)**		
Median (range)	21 (15–24)	
**FSRT Dose (Gy)**		
Median (range)	25 (24–30)	
Fractions	5 (3–5)	
**Postoperative Cavity**	7	8%
**PTV (cm**^**3**^**)**		
Median (range)	0.71 (0.012–39.1)	

Abbreviations: *PTV* Planning target volume, *SRS* Stereotactic radiosurgery, *FSRT* Fractionated stereotactic radiotherapy

### Toxicity assessments and control rates

No cases of radionecrosis were noted. Radiation-related toxicity was noted during 5 treatment sessions (16%) including grade 1–2 nausea/vomiting, headache, and fatigue. Two of these cases were managed with steroids. Prophylactic steroids were prescribed during 11 treatment sessions (35%) and steroids were continued during radiation for symptoms attributed to BCBM during 7 treatment sessions (23%). No unexpected scalp toxicities were reported during or after completion of radiation.

**Fig. 1:**
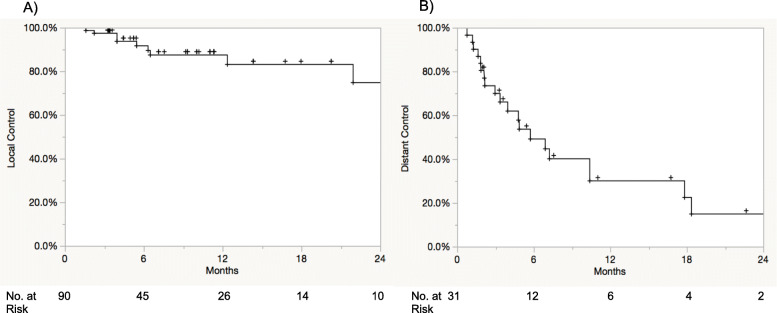
Kaplan-Meier **a**) local control and **b**) distant control following stereotactic radiation

**Fig. 2:**
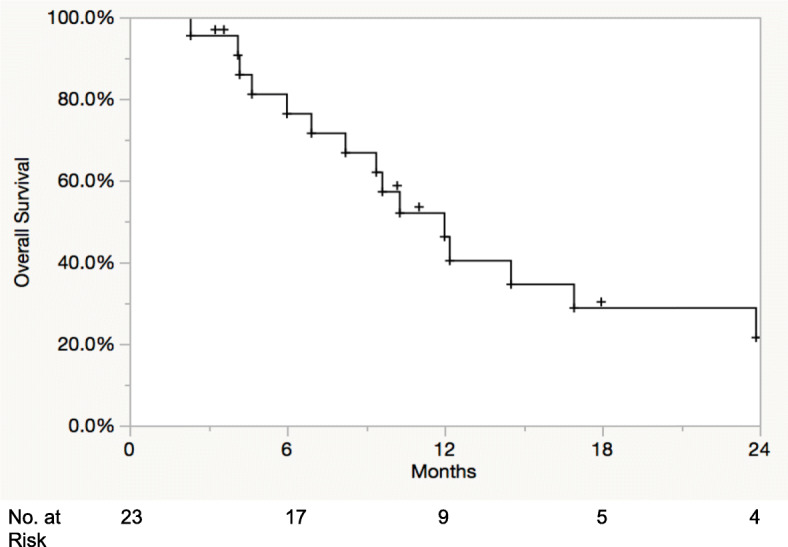
Kaplan-Meier overall survival following stereotactic radiation

Twelve- and 24-month KM LC of treated lesions was 88 and 75%, while 12- and 24-month DIC was 30 and 15%, respectively (Fig. 1a and b). No differences were noted in LC (*p* = 0.69) and DIC (*p* = 0.13) based on timing of capecitabine and stereotactic radiation. Two patients eventually developed leptomeningeal disease at 6 months and 14.1 months post SRS. Receptor types were HR+/HER2- and HR+/HER2+, respectively. From the date of stereotactic radiation, median OS was 12 months (95% CI 7–17 months), and 12- and 24-month OS were 46 and 22% (Fig. 2). From the date of brain metastasis diagnosis, median OS was 25 months (95% CI 15.3–46.6 months), and 12- and 24-month OS were 82 and 64%, respectively.

## Discussion

In this manuscript, we report our experience in the management of BCBM treated with stereotactic radiation and capecitabine. We find no increase in rates of radionecrosis, skin reactions, or other unexpected neurologic toxicities in our cohort with long-term follow-up. In addition, we note local control rates to be high while distant control remains poor consistent with experiences with stereotactic radiation alone.

Capecitabine is activated initially through hepatic metabolism and finally to 5-FU at the level of the cancer cell through the action of thymidine phosphorylase, also known as platelet-derived growth factor, which is expressed at higher levels in cancer cells than in the surrounding normal tissues [16, 17]. Radiation upregulates expression levels of thymidine phosphorylase, thus acting as a greater-than-additive interaction between radiation and capecitabine and making capecitabine a potent radiosensitizer [18]. Capecitabine and 5-FU are particularly utilized in the management of gastrointestinal malignancies [9–11] but synergistic effects with radiotherapy have also been demonstrated in the management of cervical and head and neck cancers [19, 20]. Thus, concurrent administration of capecitabine with radiation therapy should proceed with caution, particularly with high dose radiation therapy surrounding critical structures.

Capecitabine is known to have uptake in BCBM. In a study of eight surgically resected BCBM in which capecitabine was administered hours before surgical resection, measurable drug levels of capecitabine and metabolites, 5^′^-deoxy-5-fluorocytidine, 5^′^-deoxy-5-fluorouridine, and 5-fluorouracil, were detected in all resected samples [5]. Capecitabine has also been studied alongside HER TKIs neratinib and lapatinib demonstrating CNS activity [6, 7]. In addition, the recently reported HER2CLIMB study combined capecitabine with the oral TKI tucatinib and trastuzumab [21]. Among patients with brain metastases, progression-free survival (PFS) at 1 year was 25% in the tucatinib-combination group and 0% in the placebo-combination group with a 2.2 month PFS improvement.

Multiple studies have shown stereotactic radiation alone to be well tolerated. The incidence of late toxicity following radiosurgery has been reported to be 4% [22]. In RTOG 9005, following single fraction radiosurgery, the rates of radiation necrosis in surgical pathology of previously irradiated tissue were 8 and 11% at 12 and 24 months, respectively [12]. Symptomatic radionecrosis has been reported to be 10% using radiographic criteria [23]. Concurrent administration of certain systemic agents may increase the risk of radionecrosis following radiosurgery. The administration of concurrent immune checkpoint inhibitors with stereotactic radiation in melanoma, non-small cell lung cancer, and renal cell carcinoma brain metastases has been reported to potentially increase the risk of radionecrosis [24]; however, the rate of radionecrosis was not increased in prospective trials and retrospective series [25–28]. There is also not clear consensus on whether concurrent BRAF inhibition with SRS increases the risk of radionecrosis [29, 30] in melanoma brain metastases. In our series with approximately half of BCBM treated with concurrent capecitabine, we did not note any cases of radionecrosis.

Studies have shown that delivery of radiation therapy can facilitate the entry of agents into the blood-tumor barrier in the brain [31–33]. A study from Cao et al. revealed gadolinium diethylenetriaminepentaacetic acid uptake index was highest in the 30 days following whole brain radiation therapy (WBRT) [31]. In addition, the uptake index was higher across the blood-tumor barrier than the blood-brain barrier. Teng et al. analyzed 30 patients with 64 brain metastases treated with either WBRT or SRS, and similarly, 2 to 4 weeks post-treatment there was an increase in permeability for lesions with low permeability at baseline [33]. Administering capecitabine in the window following radiation may increase potential synergy. In a case series of five patients administered WBRT with capecitabine for BCBM, a complete response was noted in 1 patient and partial responses in 2 with grade 1 headaches and nausea reported in two patients each [34].

In this first reported series of capecitabine with stereotactic radiation, intracranial toxicities were similar to those expected with stereotactic radiation alone, including grade 1–2 headaches, fatigue, and nausea/vomiting noted during 15% of treatment sessions. No cases of radionecrosis were noted. Potential uptake of capecitabine following stereotactic radiation did not appear to increase the risk of toxicity. In addition, local control appears to be within the range of previously reported series, thus further data is needed to support a synergistic effect. However, distant failure continued to be a concern in our series with a poor 12-month distant control rate of 30%.

There are several important limitations to the present study, including its retrospective nature and the heterogeneity of the patient cohort. The small sample size and limited follow up due to the protracted survival of patients with BCBM limits our conclusions concerning the risk of radionecrosis.

## Conclusions

In conclusion, we note the receipt of capecitabine along with stereotactic radiation to be well tolerated without a side effect profile that appeared to be worse than stereotactic radiation alone. Although local control appears similar to previously reported series, distant control remains poor and warrants further study into novel combinations of systemic therapy along with radiotherapy to improve intracranial progression.

## Data Availability

The datasets used and/or analysed during the current study are available from the corresponding author on reasonable request.

## Abbreviations

BCBM: Breast cancer brain metastases
5-FU: 5-flourouracil
BBB: Blood-brain-barrier
TKI: Tyrosine kinase inhibitor
CNS: Central nervous system
OS: Overall survival
SRS: Stereotactic radiosurgery
FSRT: Fractionated stereotactic radiotherapy
MRI: Magnetic resonance imaging
CT: Computed tomography
GTV: Gross tumor volume
PTV: Planning target volume
IMRT: Intensity modulated radiotherapy
LC: Local brain metastases control
DIC: Distant intracranial control
KM: Kaplan–Meier
WBRT: Whole brain radiation therapy
